# Synthesis and Antitumor Evaluation of Biotin-SN38-Valproic Acid Conjugates

**DOI:** 10.3390/molecules28093936

**Published:** 2023-05-07

**Authors:** Yi Dai, Yang Zhang, Tianxiang Ye, Yue Chen

**Affiliations:** 1 College of Pharmaceutical Science, Anhui Xinhua University, Hefei 230088, China; 2 Department of General Surgery, The First Affiliated Hospital of University of Science and Technology of China, Hefei 230031, China; muxiao3000@163.com

**Keywords:** 7-ethyl-10-hydroxy-camptothecin, valproic acid, biotin, conjugate, synthesis, anticancer

## Abstract

Despite the strong anticancer activity of SN38 (7-ethyl-10-hydroxy-camptothecin), the severe side effects and loss of anticancer activity caused by the lack of selectivity to cancer cells and hydrolysis of ring E prevent its clinical application. To address the issue, herein a multifunctional SN38 derivative (compound **9**) containing biotin (tumor-targeting group) and valproic acid (histone deacetylase inhibitor, HDACi) was synthesized via click chemistry and evaluated using MTT assay. The in vitro cytotoxicity study showed that compound **9** exhibited superior cytotoxicity than irinotecan against human cervical cancer HeLa cells, albeit it was inferior to SN38. More significantly, compound **9** significantly reduced toxicity in mouse embryonic fibroblast NIH3T3 cells, indicating that compound **9** had the capacity to enhance tumor targeting due to its cell selectivity. Further studies demonstrated that, compared with irinotecan, compound **9** induced similar apoptosis of cancer cells. Consequently, compound **9** can not only improve its tumor-targeting ability mediated by biotin but also exert potent anticancer activity through the effect of SN38 and valproic acid, indicating that the design concept is an effective strategy for the structural modification of SN38.

## 1. Introduction

Since camptothecin was isolated from the bark and stem of *Camptotheca acuminata* in 1966, the alkaloid has received extensive attention from chemists and pharmacologists. Camptothecin-based compounds usually exert their anticancer effect by inhibiting DNA topoisomerase I (topo 1) [1,2,3]. Topo 1 plays an important role in the transcription and replication of cells through relieving the stress of DNA supercoiling [4]. Some camptothecin derivatives, such as topotecan and irinotecan, have been approved by the US Food and Drug Administration for the treatment of several cancers [5]. However, severe side effects due to lack of selectivity to cancer cells and loss of anticancer activity caused by hydrolysis of E-lactone is still a problem to be solved for camptothecin-based compounds, not excepting 7-ethyl-10-hydroxy-camptothecin (SN38) [6,7]. SN38, an active metabolite of irinotecan, is one of the most potent camptothecin derivatives. Though anticancer activity of SN38 is 100–1000 times stronger than that of irinotecan, only 2–8% of dosed irinotecan can be converted into SN38 to perform its therapeutic action [8]. Hence, further study on SN38 is in demand. Some research achievements on SN38 have been reported in recent years [9,10,11,12]. For example, the HSP90 inhibitor-SN38 conjugate showed excellent in vitro and in vivo antitumor activity [13]. In addition, the conjugation of SN38 with artesunate also demonstrated potent anticancer activity through the formation of reactive radical species by the cleavage of the endoperoxide bridge in artesunate and the inhibition of topo 1 by SN38 [14]. Thus, the investigation on SN38 is still a good source of developing new drugs.

Histone deacetylase (HDAC) belongs to the proteinase family and plays an important role in the structural modification of chromosomes and the regulation of gene expressions [15]. Inhibition of HDAC can suppress the growth of cancer cells. Therefore, histone deacetylase inhibitors (HDACis) have been widely explored for the treatment of cancer [16,17]. For example, belinostat and panobinostat have been approved to cure several tumors by the FDA. Chidamide was approved to cure peripheral T-cell lymphoma by the National Medical Products Administration [18,19]. Some of the literature have reported that HDACis can sensitize chemotherapeutic drugs, including camptothecin-based drugs [20,21]. For example, histone deacetylase class IIa inhibitor with lenvatinib synergistically inhibited the growth of hepatocellular carcinoma cells [22]. Panobinostat synergistically enhanced the cytotoxicity of microtubule destabilizing drugs in ovarian cancer cells [23]. In addition, hybrids of HDACis and camptothecin also exhibited potent antitumor activity in A549 and HCT-116 cells [24]. Valproic acid, a classic antiepileptic drug, also exerts its inhibitory activity against HDAC and sensitizes chemotherapeutic drugs [25,26,27]. However, a higher concentration of valproic acid is required for suppressing the growth of cancer cells through the inhibition of HDAC [27,28,29]. Therefore, the combination of valproic acid with other chemotherapeutic agents is a good strategy for the development of anticancer drugs.

The decoration of SN38 with a tumor-targeting group is an excellent strategy to reduce side effects caused by SN38. These tumor-targeting groups are usually antibodies or small molecular compounds [30,31,32,33]. For instance, trastuzumab deruxtecan, an antibody drug conjugate, was approved by the FDA to cure refractory HER2-positive metastatic breast cancer [34]. Biotin is also known as vitamin H and vitamin B7 and is responsible for various normal cellular functions. Biotin is used as a tumor-targeting agent due to the overexpression of biotin receptors on several kinds of cancer cells. The incorporation of biotin into SN38 will benefit the cell selectivity of SN38 to reduce side effects [35,36,37]. It is well established that the ester bond is one of the most commonly used linkers for the design of a prodrug and can be hydrolyzed to release a parent drug by esterase in cells. The design of a prodrug based on the ester bond was applied to the study of SN38 [24,38,39]. Based on these findings, a novel SN38 derivative containing three valproic acids (HDACis) and biotin (tumor-targeting group) (compound **9**) was synthesized as a prodrug via click chemistry under mild conditions. A cytotoxicity test showed that compound **9** had higher antiproliferative activity toward HeLa cells and a lower inhibition toward NIH3T3 cells compared with irinotecan.

## 2. Results and Discussion

### 2.1. Chemistry

**Scheme 1:**
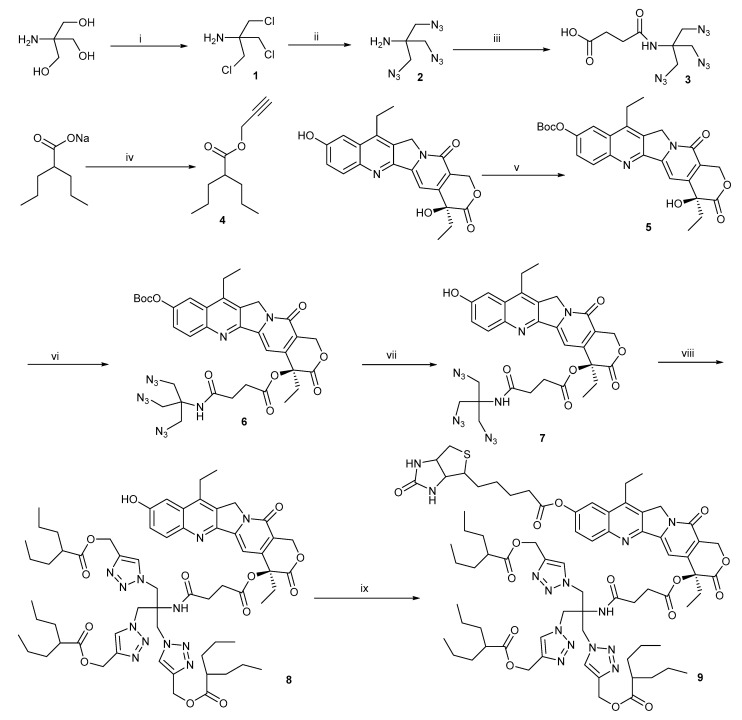
Synthetic route of compound **9**. (i) SOCl_2_, pyridine, 100 °C, 5 h, yield 63%; (ii) NaN_3_, DMSO, 90 °C, 24 h, yield 93%; (iii) succinic anhydride, triethylamine, CH_2_Cl_2_, room temperature, monitored by TLC, yield 88%; (iv) propargyl bromide, K_2_CO_3_, DMF, room temperature, monitored by TLC, yield 86%; (v) (Boc)_2_O, pyridine, CH_2_Cl_2_, room temperature, monitored by TLC, yield 86%; (vi) compound **3**, EDCI, DMAP, CH_2_Cl_2_, room temperature, monitored by TLC, yield 73%; (vii) trifluoroacetic acid, CH_2_Cl_2_, 30 °C, monitored by TLC, yield 82%; (viii) compound **4**, sodium ascorbate, CuSO_4_, t-BuOH/H_2_O mixture (2:1), 40 °C, monitored by TLC, yield 52%; (ix) biotin, EDCI, DMAP, DMF, 24 h, yield 43%.

It is well established that the opening of a lactone E-ring for camptothecin analogues will result in the decreased anticancer activity and unexpected side effects and that the lactone E-ring of compound **9** can be stabilized by the introduction of groups with a high steric hindrance C-20 of SN38 [40]. Hence, as many valproic acid moieties as possible are in need for the design of SN38 derivatives. Here, we initially selected three valproic acid moieties to conjugate with SN38 to prepare the target compound **9** for facilitating its stability. Owing to a lack of cell selectivity for SN38, biotin was used as a tumor-targeting unit of compound **9**. However, on the premise of ensuring the structural integrity of SN38, there are not enough hydroxyl groups for one molecule of SN38 to link three valproic acid molecules and one biotin molecule via an ester bond. Hence, an SN38-based dendritic compound was designed to meet the requirement of the link between five molecules (biotin; SN38; valproic acid = 1:1:3). The synthetic routes of compound **9** are described in Scheme 1. Firstly, two key intermediates, compounds **3** and **4** needed to be completed before the synthesis of compound **9**. After that, SN38 was protected with (Boc)_2_O/pyridine at C_10_-OH to prepare compound **5**, followed by esterification of compound **5** at C_20_-OH with compound **3**. The obtained compound **6** underwent the removal of the protecting group under the condition of trifluoroacetic acid and dichloromethane to synthesize compound **7**. Then, the key intermediate compound **8** was synthesized by click reaction of cu-catalytic azide-alkyne click (CuAAC) between compound **7** and compound **4** in the presence of sodium ascorbate and CuSO_4_. Finally, esterification of compound **8** and biotin was conducted under the condition of EDCI and DMAP to obtain target compound **9**. These compounds were verified by HPLC, NMR, and MS (Figures S1–S14).

### 2.2. In Vitro Biological Evaluation

#### 2.2.1. In Vitro Cytotoxicity

**Figure 1:**
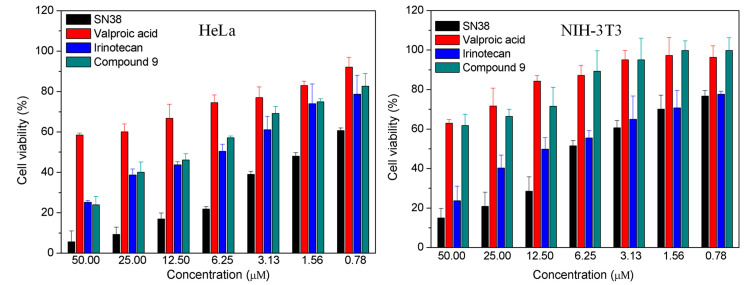
The in vitro cytotoxicity evaluated by MTT assay on HeLa cells and NIH3T3 cells. Cells were co-incubated with different concentrations of compounds for 48 h.

The biotin receptor was overexpressed in various tumor cells [41,42]. The incorporation of biotin into the chemotherapeutic agents was a good strategy for enhanced selectivity to cells [43,44,45]. The designed compound **9** was expected to possess multifunction, with one biotin molecule on one end to target cancer cells and three releasable valproic acid molecules on the other end to inhibit HDAC. Here, we chose a biotin receptor-positive cancer cell line (HeLa) and a negative-normal cell line (NIH3T3) to investigate cytotoxicity and selectivity in the cells of compound **9**. The half-maximal inhibitory concentrations (IC_50_) indicated that compound **9** was comparable to irinotecan and weaker than SN38 towards HeLa cells (Figure 1). The reason why the cytotoxicity of compound **9** was inferior to SN38 might be that compound **9** only partly released SN38 and valproic acid. Fortunately, compound **9** exhibited lower cytotoxicity than SN38 and irinotecan toward NIH3T3 cells, demonstrating that biotin moiety in compound **9** worked (Figure 1). The survival rate of NIH3T3 cells in compound **9** group was above 60% at the dose of 50 µM, suggesting that compound **9** had the potential of reducing side effects. Moreover, the IC_50_ values of SN38 and irinotecan to NIH3T3 cells were both under 12.50 µM. There was no difference in cytotoxicity for compound **8** toward HeLa cells and NIH3T3 cells at a dose of 50 µM, as shown in Figure S15. In addition, valproic acid and biotin both showed only marginal cytotoxicity against HeLa cells and NIN3T3 cells (Figure S16). These findings demonstrated that the combination of SN38 with valproic acid and biotin not only retained anticancer activity similar to irinotecan but also enhanced the selectivity to cells.

#### 2.2.2. Mitochondrial Membrane Potential Analysis

**Figure 2:**
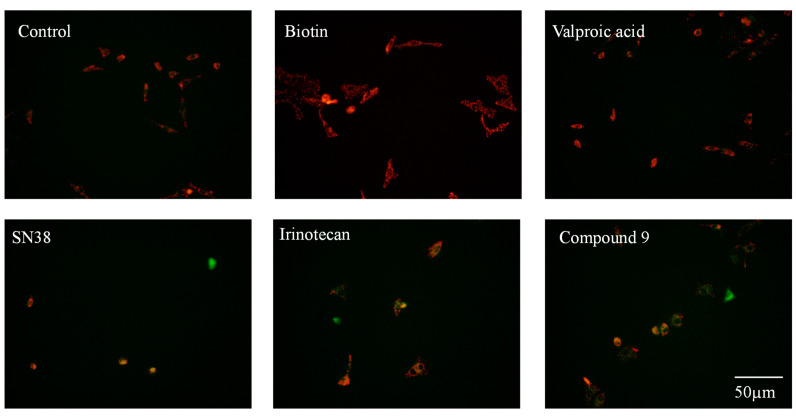
Mitochondrial membrane potential analyzed by fluorescence microscope. HeLa cells were co-incubated with 6 µM of valproic acid or 2 µM of SN38, irinotecan, and compound **9** for 24 h, stained with JC-1, and detected by fluorescence microscope.

Apoptosis is a major pathway of camptothecin analogues for inhibiting the growth of cancer cells [46,47,48]. Mitochondria play a key role in the apoptosis of cells. The decreased mitochondrial membrane potential (∆Ψm) is a hallmark event in the early stage of cell apoptosis. The cell mitochondrial membrane potential can be detected by using the JC-1 staining method. JC-1 can aggregate in normal mitochondria and present red fluorescence. Once the ∆Ψm decreases, JC-1 presents green fluorescence due to the dispersion of aggregates; the smaller the ratio of red and green fluorescence, the more the ∆Ψm drops. The results of the JC-1 staining method showed that, compared with the control, valproic acid hardly decreased ∆Ψm at a dose of 6 µM, while SN38, irinotecan, and compound **9** significantly decreased ∆Ψm at a dose of 2 µM (Figure 2 and Figure S17). The ability of compound **9** to decrease ∆Ψm was comparable to that of irinotecan and weaker than that of SN38, consistent with the results of the cytotoxicity assay. These findings demonstrated that compound **9** inhibited the growth of cancer cells through the same mitochondria-mediated apoptosis as other camptothecin derivatives.

#### 2.2.3. Apoptosis Study

**Figure 3:**
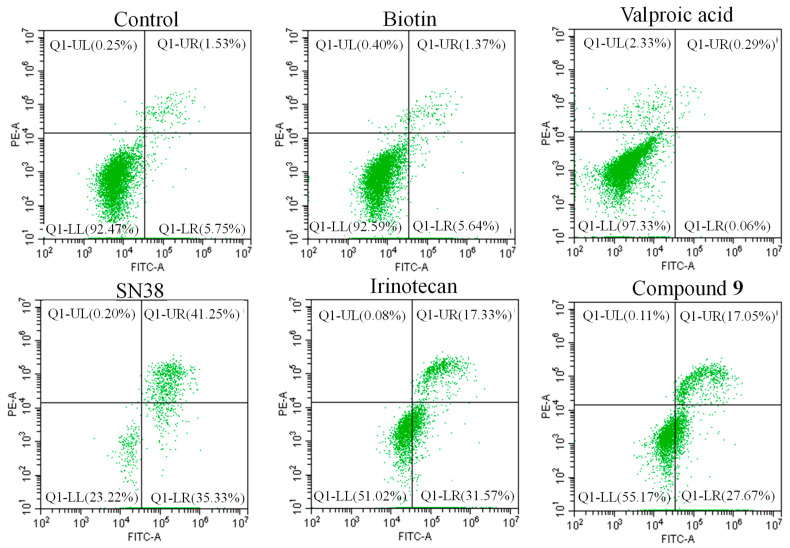
Cell apoptosis detected by flow cytometry. HeLa cells were co-incubated with 6 µM of valproic acid or 2 µM of SN38, irinotecan, and compound **9** for 48 h, stained with Annexin V-FITC/PI and detected by flow cytometry.

Based on the above findings regarding the decrease in mitochondrial membrane potential caused by compound **9**, apoptosis induced by compound **9** was analyzed using flow cytometry. As shown in Figure 3, valproic acid and biotin at a dose of 2 µM did not effectively induce apoptosis. Compared with the control group, the percentage of apoptotic cells in the valproic acid group and the biotin group was negligible (<8%). SN38 still exhibited the strongest ability to induce apoptosis among the three tested camptothecin derivatives and induced a 76.6% apoptotic rate toward the HeLa cells. Although compound **9** could not induce a strong apoptosis similar to SN38, it induced a 44.7% apoptotic rate toward the HeLa cells. Similarly, there was no significant difference in apoptosis induced by compound **9** and irinotecan, consistent with the cell proliferation assay findings. The research on inducing apoptosis showed that compound **9** exerted an antitumor effect mainly by inducing apoptosis of cancer cells.

## 3. Materials and Methods

SN38 and valproic acid were purchased from Energy Chemical (Anhui Zesheng Technology Co., Ltd., Anqin, China). Other chemical reagents were obtained from Sinopharm Chemical Reagent Co., Ltd. (Sinopharm Group Co. Ltd., Shanghai, China). Compound **9** was dissolved in 0.05% DMSO. Thin-layer chromatography on 0.25 mm silicon gel plates (GF254) were purchased from Energy Chemical Co., Ltd. DEME culture medium was purchased from Hyclone. (Thermo Fisher Scientific, Logan, UT, USA). FBS was purchased from Gibco (Thermo Fisher Scientific, Logan, UT, USA). MTT, JC-1 probe, and apoptosis detection kits were purchased from Beyotime Biotechnology (Biyuntian Co., Ltd., Shanghai, China). Murine fibroblast NIH3T3 cells and human cervical cancer HeLa cells were purchased from ATCC of US. NMR spectra were recorded on Bruker DRX-300 NMR and Bruker DRX-400 NMR (Bruker Co., Bremen, Germany). Mass spectra (MS) were obtained using an Agilent LC-MS (ESI) (Agilent Co., Santa Clara CA, USA). Fluorescence imaging was observed on an Olympus IX71 fluorescence microscope (Olympus Co., Tokyo, Japan). Apoptosis was analyzed on CytoFLEX flow cytometry (Bechman Coulter, Pasadena, CA, USA).

### 3.1. Synthesis of Compound ***9***

#### 3.1.1. The Synthetic Route of Compound **1**

Compound **1** was synthesized as described in the literature [49]. In brief, tris (15 g, 0.124 mol) and SOCl_2_ (39 mL, 0.519 mol) were placed in a 250 mL flask and stirred at 0 °C. After pyridine (5 mL, 0.062 mol) was added dropwise to the reaction solution, the reaction temperature was raised slowly to 100 ℃. The reaction solution was stirred at 100 °C for 5 h, followed by another 1 h of reaction at 120 °C. Then, the reaction solution was cooled to 20 °C and treated with 30 mL H_2_O. The pH value of the aqueous solution was adjusted to 10 with sodium carbonate solution. The obtained aqueous solution was extracted with dichloromethane three times. The organic layer was dried with anhydrous sodium sulfate. After the removal of dichloromethane in vacuo, the obtained mixture was purified using silica gel chromatograph with petroleum ether as the elution solvent. The pure compound **1** was a colorless liquid with a yield of 63%. ^1^H-NMR (300 MHz, Chloroform-*d*) δ 3.67 (s, 6H), 1.73 (s, 2H).

#### 3.1.2. The Synthetic Route of Compound **2**

Compound **2** was synthesized as described in the literature [50]. Compound **1** (3 g, 0.017 mol) and NaN_3_ (3.65 g, 0.056 mol) were dissolved in 25 mL DMSO and stirred for 24 h at 90 °C. Then, the reaction solution was cooled to 20 °C and poured into 250 mL H_2_O. The obtained solution was extracted with diethyl ether (3 × 80 mL). The organic layer was washed with brine and H_2_O and dried with anhydrous magnesium sulphate. After the removal of diethyl ether in vacuo, compound **2** was obtained as a colorless oil with a yield of 93%. ^1^H-NMR (300 MHz, Chloroform-*d*) δ 3.34 (s, 6H), 1.53 (s, 2H).

#### 3.1.3. The Synthetic Route of Compound **3**

To a solution of compound **2** (1.57 g, 8 mmol) in 10 mL dichloromethane, triethylamine (1 mL) and succinic anhydride (0.8 g, 8 mmol) were added to react at room temperature. The reaction process was monitored using thin layer chromatography (TLC). After the reaction was completed, the reaction solution was washed with saturated KHSO_4_ and brine. The organic layer was dried with anhydrous magnesium sulphate. After the removal of dichloromethane in vacuo, compound **3** was obtained as a white solid with a yield of 88%. ^1^H-NMR (300 MHz, Chloroform-*d*) δ 5.91 (s, 1H), 3.70 (s, 6H), 2.71 (t, *J* = 6.6 Hz, 2H), 2.52 (t, *J* = 6.6 Hz, 2H).

#### 3.1.4. The Synthetic Route of Compound **4**

Compound **4** was synthesized in accordance with the literature [51]. Sodium valproate (6.64 g, 40 mmol), propargyl bromide (7.14 g, 60 mmol), and K_2_CO_3_ (1.6 g, 12 mmol) were dissolved in 25 mL DMF and stirred at room temperature. The reaction process was monitored by TLC. After the reaction was completed, the reaction solution was poured into 100 mL H_2_O and extracted with ethyl acetate. The organic layer was washed with brine and H_2_O, respectively, and dried with anhydrous sodium sulfate. After the removal of ethyl acetate in vacuo, compound **4** was obtained as a white solid with a yield of 86%. ^1^H-NMR (400 MHz, Chloroform-*d*) δ 4.68 (d, *J* = 2.5 Hz, 2H), 2.48 (t, *J* = 2.5 Hz, 1H), 2.45–2.35 (m, 1H), 1.72–1.53 (m, 2H), 1.50–1.38 (m, 2H), 1.37–1.19 (m, 4H), 0.90 (t, *J* = 7.3 Hz, 6H).

#### 3.1.5. The Synthetic Route of Compound **5**

Compound **5** was synthesized as described in the literature [52]. The mixture of SN38 (1.00 g, 2.50 mmol), (Boc)_2_O (1.00 g, 4.50 mmol), pyridine (5 mL), and dichloromethane (50 mL) was stirred in a flask at room temperature. The reaction process was monitored using TLC. After the reaction was completed, the reaction solution was washed three times with 1% HCl and water, respectively. The resulting dichloromethane fraction was dried with anhydrous magnesium sulfate, filtered, and concentrated in vacuum to obtain compound **5**, a faint yellow solid with a yield of 86%. ^1^H-NMR (300 MHz, Chloroform-*d*) δ 8.26 (d, *J* = 9.2 Hz, 1H), 7.91 (d, *J* = 2.5 Hz, 1H), 7.78–7.56 (m, 2H), 5.77 (d, *J* = 16.4 Hz, 1H), 5.44–5.16 (m, 3H), 3.84 (s, 1H), 3.18 (q, *J* = 7.6 Hz, 2H), 2.07–1.80 (m, 2H), 1.63 (s, 9H), 1.42 (t, *J* = 7.6 Hz, 3H), 1.05 (t, *J* = 7.4 Hz, 3H). Analytical data were in suitable accordance with the reported data [52]. Judging from the signal of 1.57ppm in NMR, some Boc-OH existed in the product. Since Boc-OH had difficulty in reacting with compound **5** due to its high steric hindrance, compound **5** was directly used in the next step without further purification.

#### 3.1.6. The Synthetic Route of Compounds **6** and **7**

Compounds **6** and **7** were synthesized by the method reported in the literature [53]. Compound **3** (355 mg, 1.2 mmol), compound **5** (518 mg, 1.0 mmol), EDCI (229 mg, 1.2 mmol), and DMAP (15 mg,0.12 mmol) were dissolved in 60 mL dichloromethane and stirred at room temperature. The reaction process was monitored using TLC. After the reaction was over, the reaction solution was washed three times with 1% HCl and brine, respectively. The resulting dichloromethane fraction was dried with anhydrous sodium sulfate, filtered, and concentrated in vacuum to obtain compound **6**, a faint yellow solid with a yield of 73%. The resulting compound **6,** without any purification, and trifluoroacetic acid (9 mL) were dissolved in 20 mL dichloromethane and stirred at 30 °C to remove the Boc protecting group. The reaction process was monitored using TLC. After the reaction was finished, the dichloromethane layer was washed with saturated NaHCO_3_, brine, and H_2_O, respectively. Then, the organic layer was dried with anhydrous sodium sulfate. After the removal of dichloromethane in vacuo, compound **7** was obtained as a faint yellow solid with a yield of 82%. Compound **7** was directly used in the next step without further purification. ^1^H-NMR (400 MHz, Chloroform-*d*) δ 8.10 (s, 1H), 7.44 (d, *J* = 10.0 Hz, 1H), 7.38 (d, *J* = 2.4 Hz, 1H), 7.26 (s, 1H), 5.71 (d, *J* = 17.0 Hz, 2H), 5.39 (d, *J* = 17.0 Hz, 1H), 5.19 (s, 1H), 3.67–3.44 (m, 6H), 3.03 (m, 2H), 2.89 (m, 2H), 2.66–2.47 (m, 2H), 2.30–2.05 (m, 2H), 1.33 (t, *J* = 7.7 Hz, 3H), 1.01 (t, *J* = 7.5 Hz, 3H).

#### 3.1.7. The Synthetic Route of Compound **8**

To a solution of compound **4** (300 mg, 1.65 mmol) in a t-BuOH/H_2_O mixture (2:1, 20 mL), sodium ascorbate (594 mg, 0.3 mmol) and CuSO_4_ (750 mg, 0.3 mmol) were added and stirred at room temperature and under an argon atmosphere. When this mixture was yellow, compound **7** (268 mg, 0.4 mmol) was added and stirred at 40 °C. The reaction process was monitored by TLC. After the reaction was over, t-BuOH was removed in vacuum and the residue was lyophilized to prepare the crude compound **8**. Then, the crude compound **8** was purified using silica gel column chromatography. The final compound **8** was a faint yellow solid with a yield of 52%. HPLC/Purity: 97.3% (t_R_ = 7.056), ^1^H-NMR (400 MHz, DMSO-*d*_6_) δ 10.30 (s, 1H), 8.39 (s, 1H), 8.01 (s, 2H), 7.93 (d, *J* = 8.9 Hz, 1H), 7.77 (d, *J* = 9.0 Hz, 1H), 7.34 (t, *J* = 13.5 Hz, 2H), 7.10 (s, 1H), 5.50 (s, 2H), 5.24 (d, *J* = 17.0 Hz, 2H), 5.07 (m, 6H), 4.52 (q, *J* = 8.8 Hz, *J* = 14.7 Hz, 6H), 3.14–2.98 (m, 2H), 2.89–2.60 (m, 2H), 2.40–2.23 (m, 4H), 2.15 (m, 2H), 1.55–1.21 (m, 15H), 1.20–1.07 (m, 13H), 0.95 (t, *J* = 7.1 Hz, 3H), 0.75 (t, *J* = 7.2 Hz, 18H); ^13^C NMR (101 MHz, DMSO-*d*_6_) δ 175.63, 173.14, 172.12, 167.83, 157.23, 157.05, 149.05, 147.15, 146.19, 144.01, 143.19, 142.15, 131.76, 128.59, 128.21, 127.29, 122.88, 118.04, 105.17, 94.92, 76.61, 66.61, 59.39, 57.10, 49.76, 49.06, 44.69, 34.50, 34.32, 30.58, 28.73, 22.71, 20.41, 14.19, 13.80, 8.10; ESI-MS: *m*/*z*: calcd.1239.62 ([M + Na]^+^), 1255.59 ([M + K]^+^); found 1239.70 [M + Na]^+^, 1255.65 [M + K]^+^. The peak at 8.39 ppm (s, 1H) showed active hydrogen on the amide bond. Due to hydrogen exchange, the integration of active hydrogen was insufficient. In addition, the peak splitting of hydrogen seemed to be irregular, perhaps due to the different 3D structures caused by these crowded repeating units.

#### 3.1.8. The Synthetic Route of Compound **9**

Compound **8** (1.22 g, 1 mmol), biotin (44.8 mg, 2 mmol), EDCI (0.488 g, 2.5 mmol), and DMAP (305 mg, 2.5 mmol) were dissolved in 10 mL DMF and stirred overnight at 25 °C. After the reaction was completed, 100 mL ethyl acetate was added to obtain a light yellow solution. The resulting solution was washed with brine three times and dried with anhydrous sodium sulfate. After the removal of ethyl acetate in vacuo, the crude product was obtained. Then, the crude product was purified with silica gel column chromatography to obtain a light yellow compound **9** with a yield of 43%. HPLC/Purity: 99.2% (t_R_ = 13.359). ^1^H-NMR (400 MHz, DMSO-*d*_6_) δ 8.40 (s, 1H), 8.23 (s, 1H), 8.21 (s, 1H), 8.13 (d, *J* = 9.1 Hz, 1H), 8.04 (s, 2H), 7.97 (d, *J* = 2.0 Hz, 1H), 7.80 (d, *J* = 4.8Hz, 1H), 7.60 (dt, *J* = 7.8 Hz, *J* = 2.3 Hz, 1H), 7.21 (d, *J* = 17.1 Hz, 1H), 6.51 (s, 1H), 6.41 (s, 1H), 5.52 (s, 2H), 5.37–5.16 (m, 4H), 5.10 (m, 5H), 4.98–4.77 (m, 1H), 4.62–4.48 (m, 4H), 4.40–4.29 (m, 1H), 4.23–4.13 (m, 1H), 3.22–3.10 (m, 3H), 2.86 (dd, *J* = 12.5, 5.0 Hz, 2H), 2.70 (t, *J* = 7.2 Hz, 3H), 2.61 (d, *J* = 12.4 Hz, 1H), 2.51 (dt, *J* = 8.9, 3.8 Hz, 2H), 2.38–2.24 (m, 4H), 2.22–2.09 (m, 2H), 1.73–1.25 (m, 3H), 1.63–1.21(m, 17H), 1.20–1.06 (m, 12H), 1.01–0.89 (m, 3H), 0.83–0.63 (m, 18H). ^13^C NMR (101 MHz, DMSO-*d*_6_) δ 175.64, 175.33, 173.16, 172.26, 172.14, 167.75, 163.21, 156.99, 152.17, 149.68, 146.86, 146.61, 146.15, 145.77, 142.17, 141.75, 11.50, 128.76, 127.48, 127.29, 126.14, 124.73, 119.05, 115.67, 95.77, 76.56, 66.64, 62.89, 61.51, 59.70, 59.40, 57.12, 55.82, 49.87, 49.10, 44.71, 34.51, 34.47, 34.33, 33.85, 30.61, 28.50, 28.45, 24.78, 22.68, 20.43, 20.36, 14.10, 8.11; ESI-MS: *m*/*z*: calcd.1465.69 ([M + Na]^+^), 1481.67 ([M + K]^+^); found 1465.80 [M + Na]^+^, 1481.80 [M + K]^+^. The peaks at 8.40 ppm (s, 1H), 8.23 (s, 1H), and 8.21 (s, 1H) showed active hydrogens on the amide bonds. Due to the hydrogen exchange, the integration of active hydrogens was insufficient. In addition, the peak splitting of hydrogen seemed to be irregular, perhaps due to the different 3D structures caused by these crowded repeating units.

### 3.2. In Vitro Biological Evaluation

#### 3.2.1. In Vitro Cytotoxicity Assay

Cells were dispersed in the DMEM containing 10% FBS and seeded in 96-well plates at the density of 3000 cells/well. After 12 h incubation at 37 °C in 5% CO_2_, cells were treated with 100 µL fresh medium containing varying concentrations of drugs and co-incubated for 48 h. Then, the medium containing the drugs was replaced with a fresh culture medium containing 1 mg/mL MTT, followed by incubation for 4 h. Finally, the medium containing MTT was removed completely and 150 µL of DMSO was added to every well. After shaking for 10 min at 37 °C, the absorbance was measured at 490 nm using a Bio-Rad 680 microplate reader. The IC_50_ values were calculated using GraphPad Prism software (version 5.01) based on data from three parallel experiments.

#### 3.2.2. Mitochondrial Membrane Potential Assay

Mitochondrial membrane potentials were assayed in accordance with the instructions of JC-1 Kit. Briefly, HeLa cells were seeded in 6-well plates at a density of 50,000 cells per well and incubated overnight at 37 °C in 5% CO_2_. The next day, the medium was replaced with a fresh medium containing 2 µM of these tested compounds and the cells were co-incubated with these compounds for 24 h. After the medium containing the drugs was removed, the cells were rinsed three times with PBS and treated with 1 mL JC-1 staining solution for further incubation of 20 min. Then, the cells were washed two times with buffers provided in the JC-1 kit, followed by the treatment with 1 mL serum-free medium. The fluorescence imaging of HeLa cells was monitored by determining fluorescent emissions from mitochondrial JC-1 monomers or aggregates using an Olympus fluorescence microscope. Mitochondrial membrane potentials were analyzed through fluorescence intensity.

#### 3.2.3. Cell Apoptosis Assay

Cell apoptosis was assayed in accordance with the instructions of the Annexin V-FITC apoptosis detection kit. Briefly, HeLa cells were seeded in 6-well plates at the density of 100,000 cells per well and incubated overnight at 37 °C in 5% CO_2_. The next day, the cells were treated with 2 µM of these tested compounds. After 48 h of incubation, the medium was harvested and the cells were washed with PBS, followed by trypsinization. Then, the cells were resuspended in the resulted medium and centrifugated to harvest these cells. Finally, the cells were processed as described in the AnnexinV-FITC apoptosis detection kit (Beyotime Biotechnology, Nanjing, China). The samples were assayed with a CytoFLEX flow cytometer (Beckman Coulter, Pasadena, CA, USA).

#### 3.2.4. Statistical Analysis

All data are presented as means ±SD for three independent experiments. The IC _50_ value was calculated by GraphPad Prism 5.0 (GraphPad Software, San Diego, CA, USA).

## 4. Conclusions

In this study, a novel biotin-SN38-valproic acid conjugate, compound **9,** was synthesized via click reaction. Compound **9** was a multifunctional prodrug due to the linker of ester between biotin, SN38, and valproic acid. The introduction of valproic acid at C_20_-OH of SN38 enhanced the stability of the E-ring to diminish the inactivation of SN38, while attaching SN38 to biotin could efficiently deliver SN38 into tumor tissues to reduce the harm to normal cells. The results of the MTT assay, the mitochondria membrane potential assay, and the apoptosis detection all showed that compound **9** exhibited excellent anticancer activity comparable to that of irinotecan. Moreover, compound **9** had lower cytotoxicity toward normal cells, indicating that compound **9** possessed good selectivity for cancer cells. These findings indicated that the combination of SN38 with biotin and valproic acid was an effective strategy to design novel camptothecin-based therapeutic agents.

## Data Availability

The data presented in this study are shown in this paper.

## Supplementary Materials

The following supporting information can be downloaded at: https://www.mdpi.com/article/10.3390/molecules28093936/s1, Figures S1–S14: NMR, HPLC and ESI-MS spectrum of compound **1**–**9**; Figure S15: Effects of compound **8** on cell viability of HeLa cells and NIH3T3 cells; Figure S16: Effects of biotin on cell viability; Figure S17: Effects of the tested compounds on mitochondrial membrane potentials.
