# Supplementary material for: Synthesis and Antitumor Evaluation of Biotin-SN38-Valproic Acid Conjugates
Source: Molecules. 2023 May 7;28(9):3936. doi: 10.3390/molecules28093936 (PMC10179906; doi:10.3390/molecules28093936)
Supplement: Supplementary file 1 [file molecules-28-03936-s001.zip › molecules-2391521-supplementary.pdf]

Supporting Information

Synthesis and Antitumor Evaluation of Biotin-SN38-Valproic Acid Conjugates

Yi Dai [1,*], Yang Zhang [2], Tianxiang Ye [1] and Yue Chen [1]

1 College of Pharmaceutical Science, Anhui Xinhua University, Hefei 230088, China

2 Department of General Surgery, The First Affiliated Hospital of University of Science and Technology of China, Hefei 230031, China

* Correspondence: daiyiii@163.com

Contents

Figure S1. ^{1}H NMR spectrum of compound **1**……3

Figure S2. ^{1}H NMR spectrum of compound **2**……3

Figure S3. ^{1}H NMR spectrum of compound **3**……4

Figure S4. ^{1}H NMR spectrum of compound **4**……4

Figure S5. ^{1}H NMR spectrum of compound **5**……5

Figure S6. ^{1}H NMR spectrum of compound **7**……5

Figure S7. ^{1}H NMR spectrum of compound **8**……6

Figure S8. ^{13}C-NMR spectrum of compound **8**……6

Figure S9. ESI-MS spectrum of compound **8** ……7

Figure S10. HPLC spectrum of compound **8** ……7

Figure S11. ^{1}H NMR spectrum of compound **9**……8

Figure S12. ^{13}C NMR spectrum of compound **9**……8

Figure S13. ESI-MS spectrum of compound **9**……9

Figure S14. HPLC spectrum of compound **9**……9

Figure S15. Effects of compound **8** on cell viability of HeLa cells and NIH3T3 cells……10

Figure S16. Effects of biotin on cell viability……11

Figure S17. Effects of the tested compounds on mitochondrial membrane potentials……12

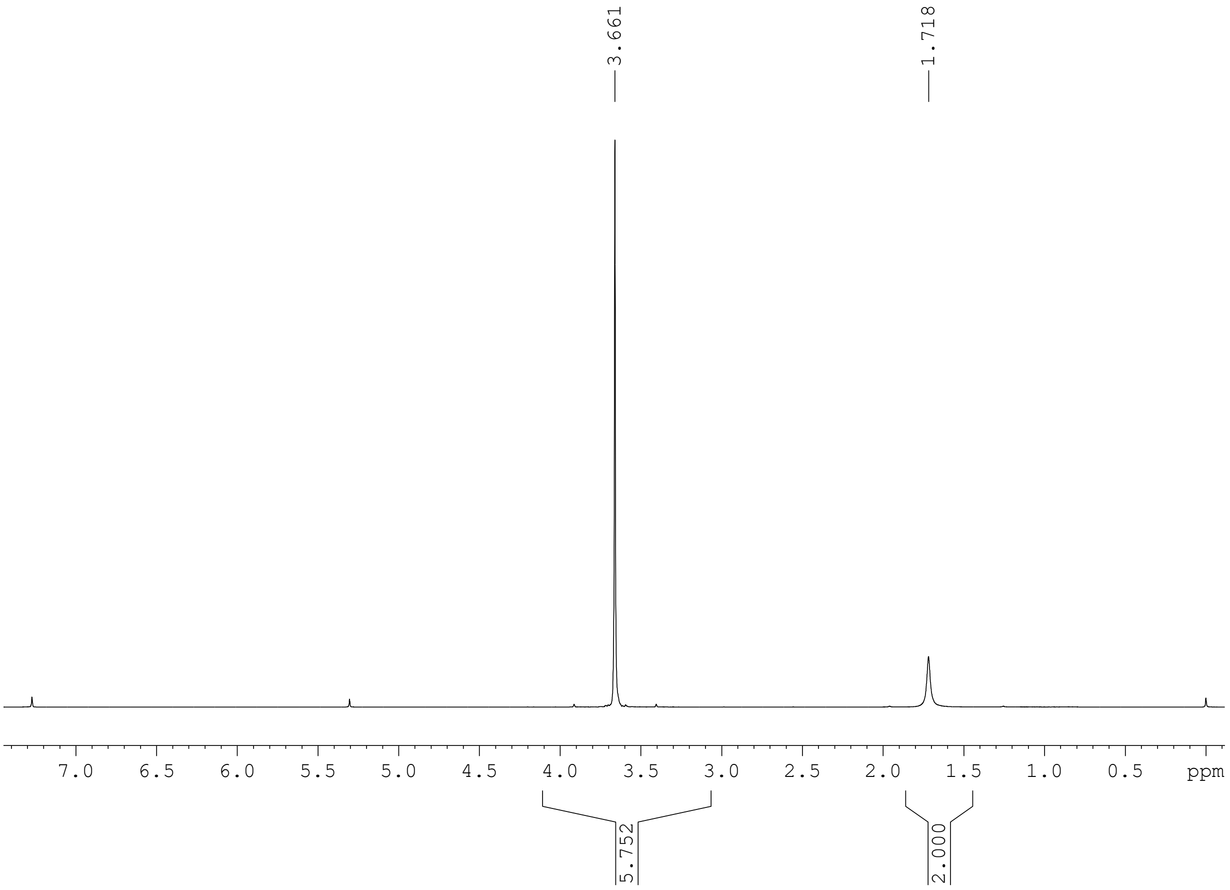

Figure S1. ^{1}H NMR spectrum of compound **1** (300 MHz, Chloroform-*d*).

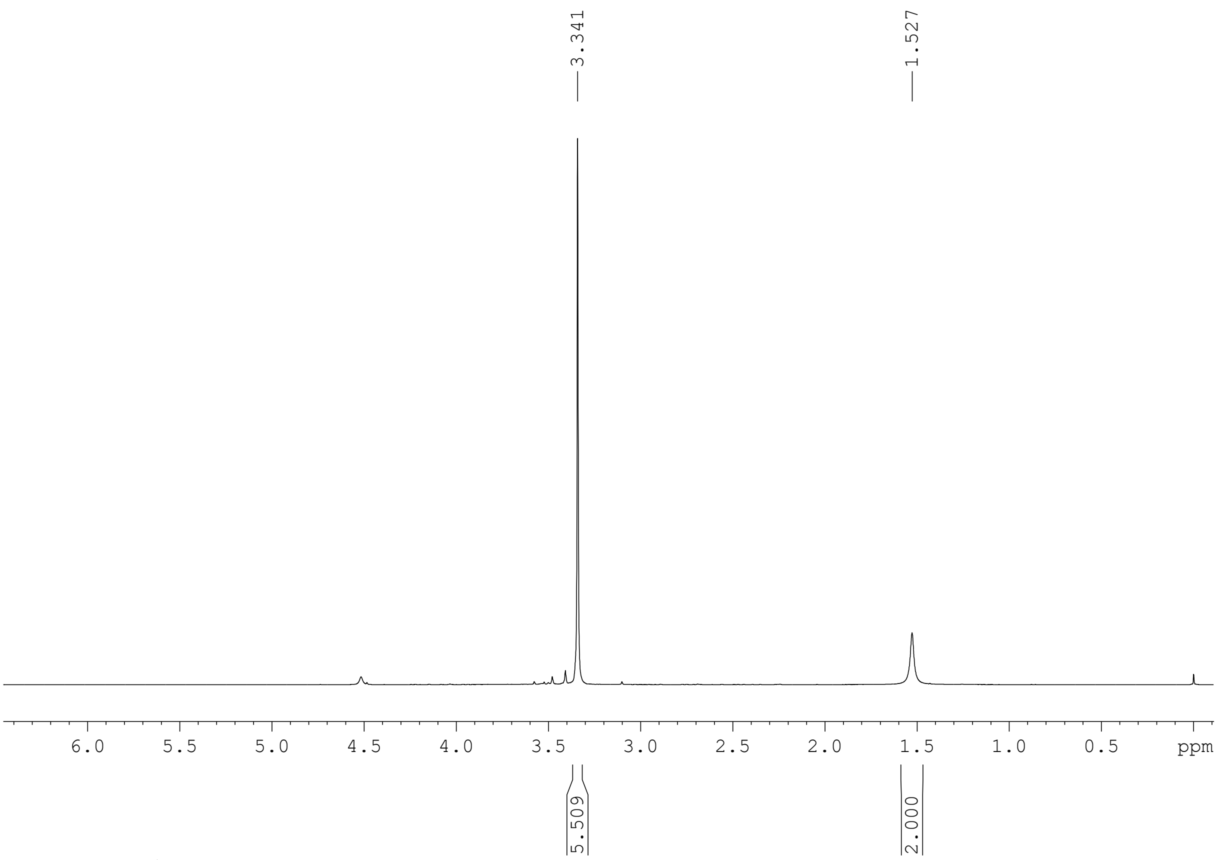

Figure S2. ^{1}H NMR spectrum of compound **2** (300 MHz, Chloroform-*d*).

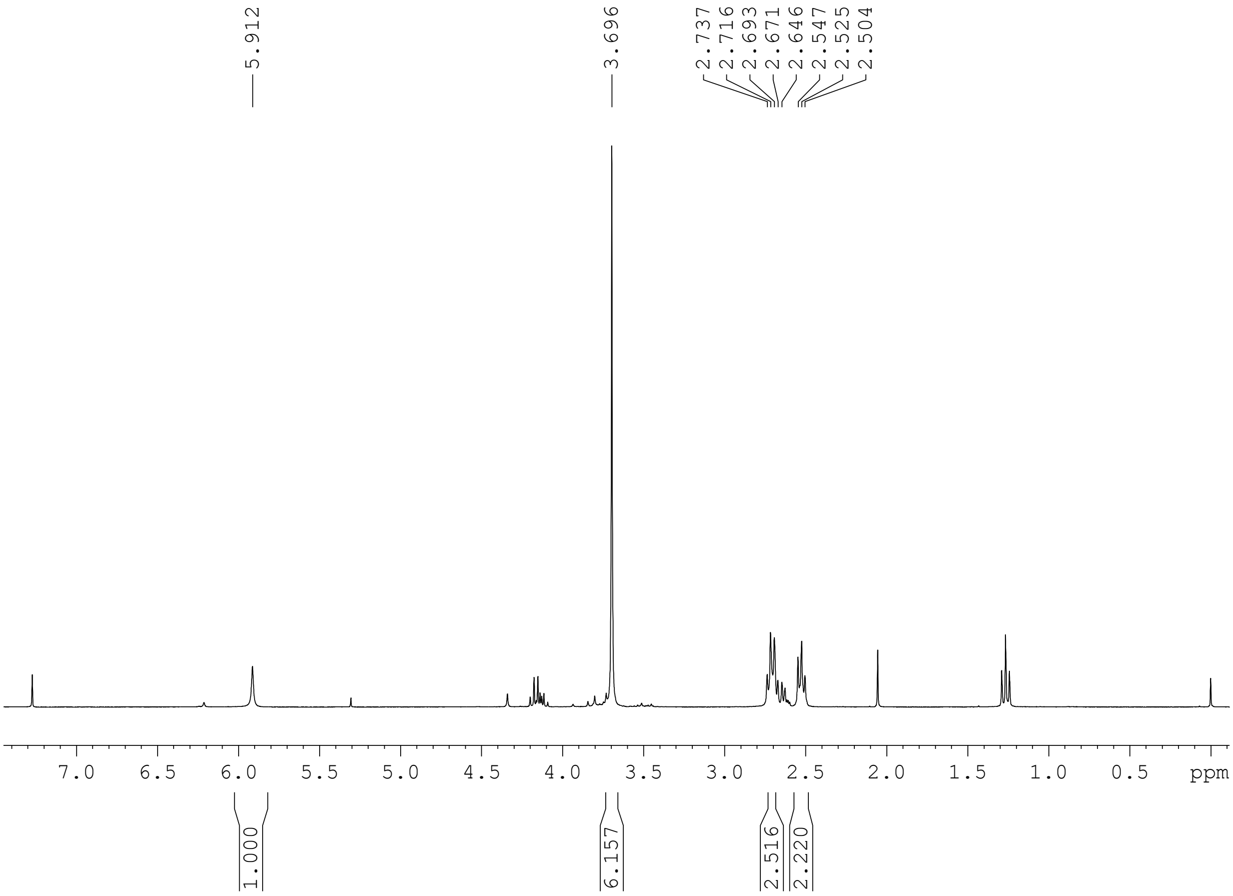

Figure S3. ^{1}H NMR spectrum of compound **3** (300 MHz, Chloroform-*d*).

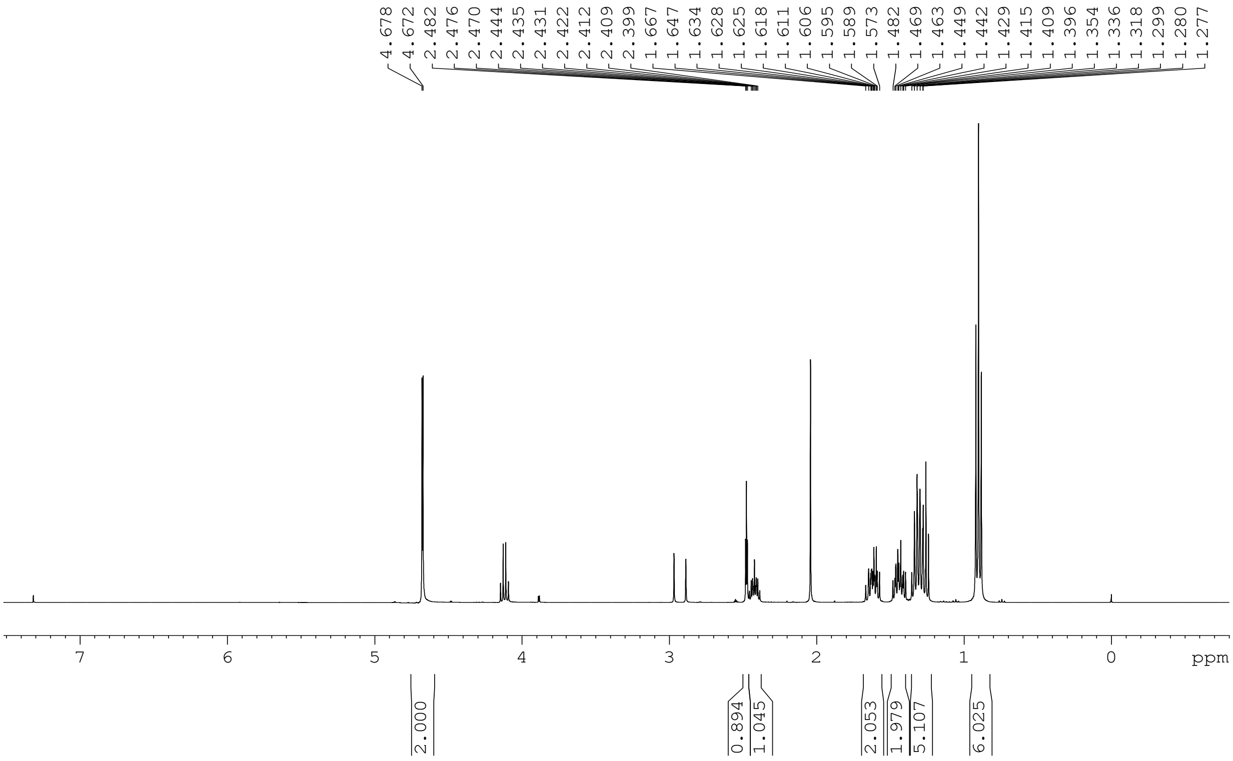

Figure S4. ^{1}H NMR spectrum of compound **4** (400 MHz, Chloroform-*d*).

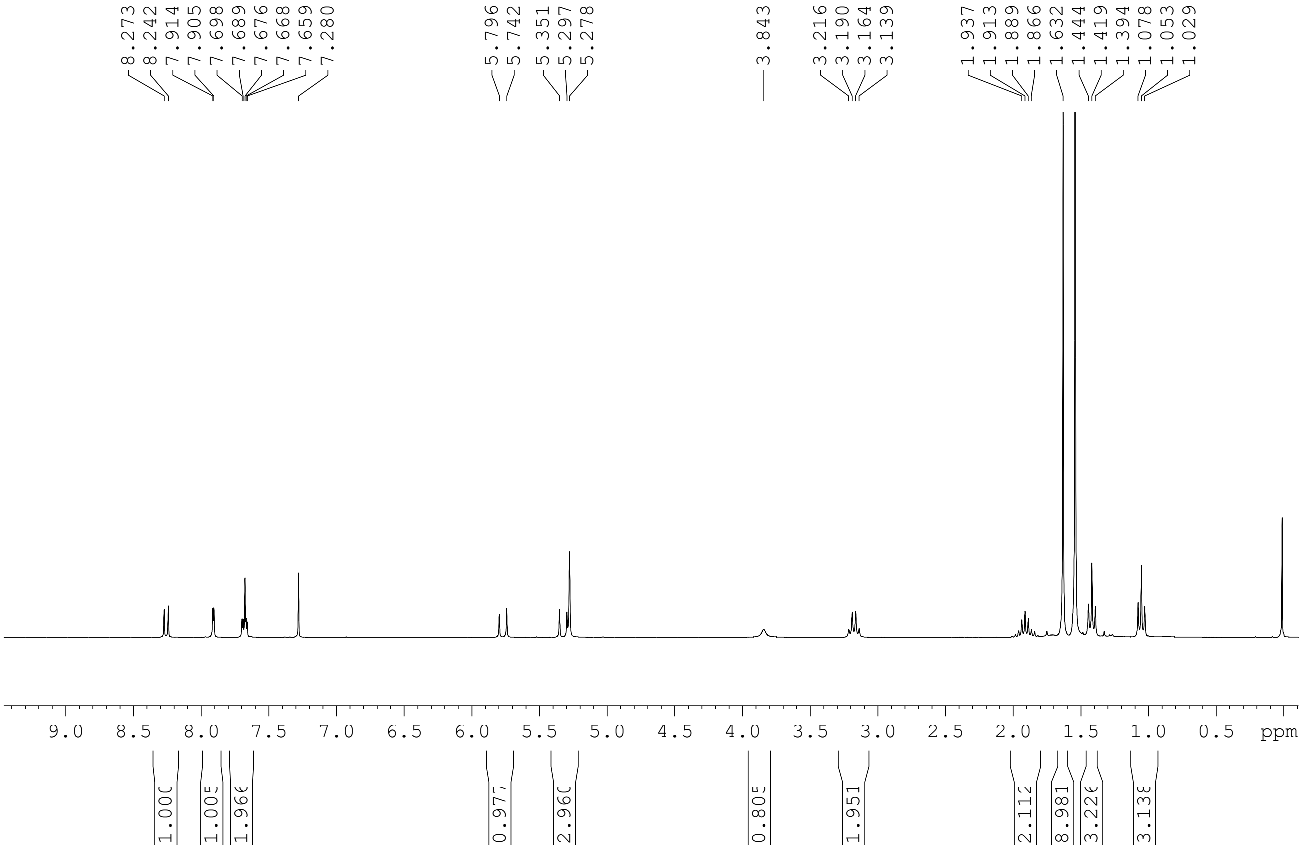

Figure S5. ^{1}H NMR spectrum of compound **5** (300 MHz, Chloroform-*d*).

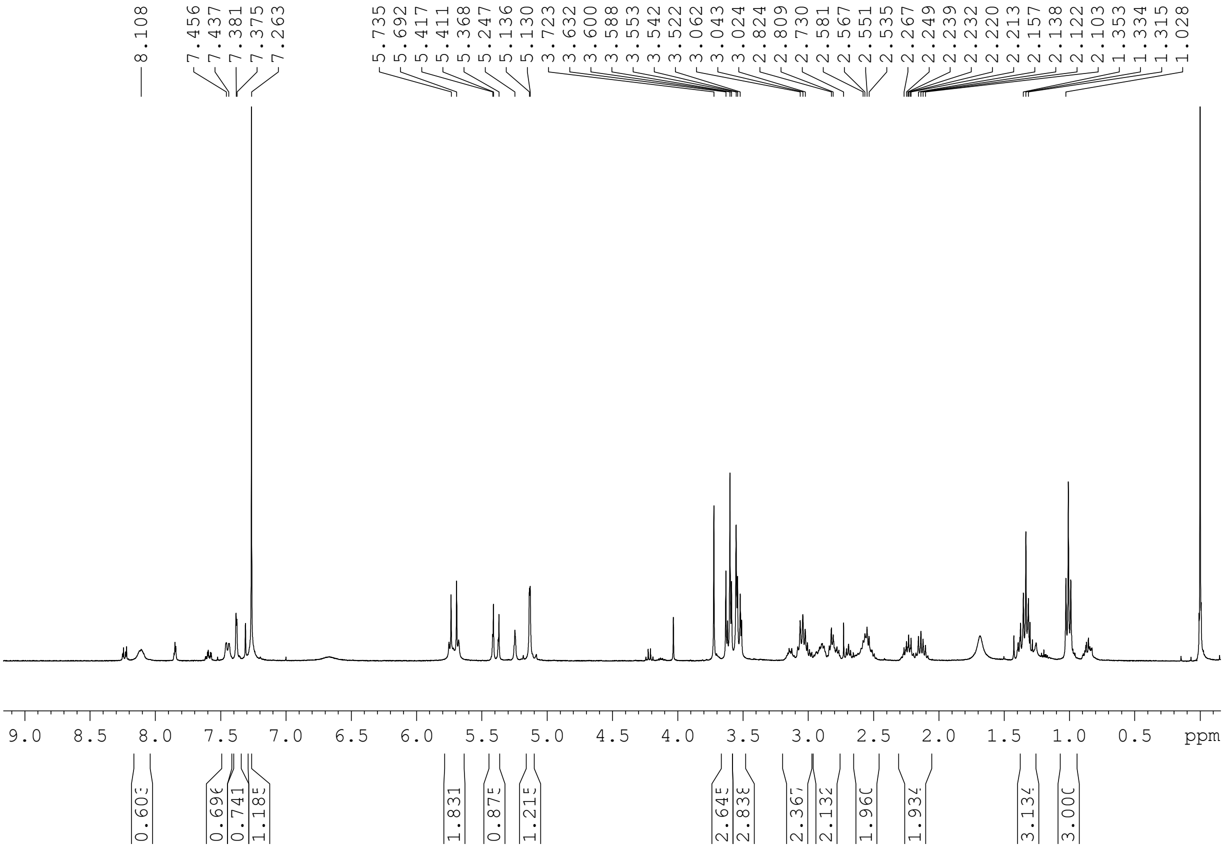

Figure S6. ^{1}H NMR spectrum of compound **7** (400 MHz, Chloroform-*d*).

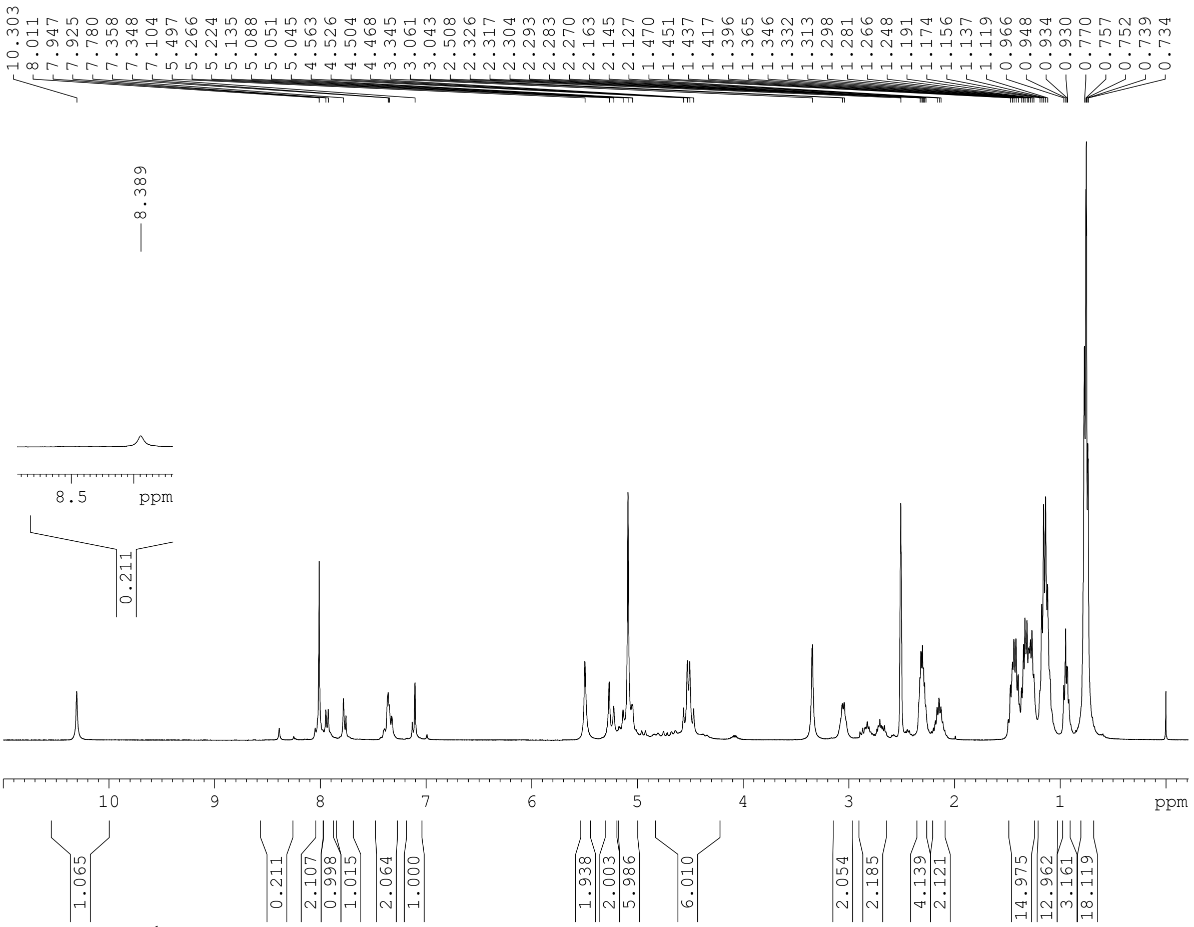

Figure S7. ^{1}H NMR spectrum of compound **8** (400 MHz,DMSO-d_6).

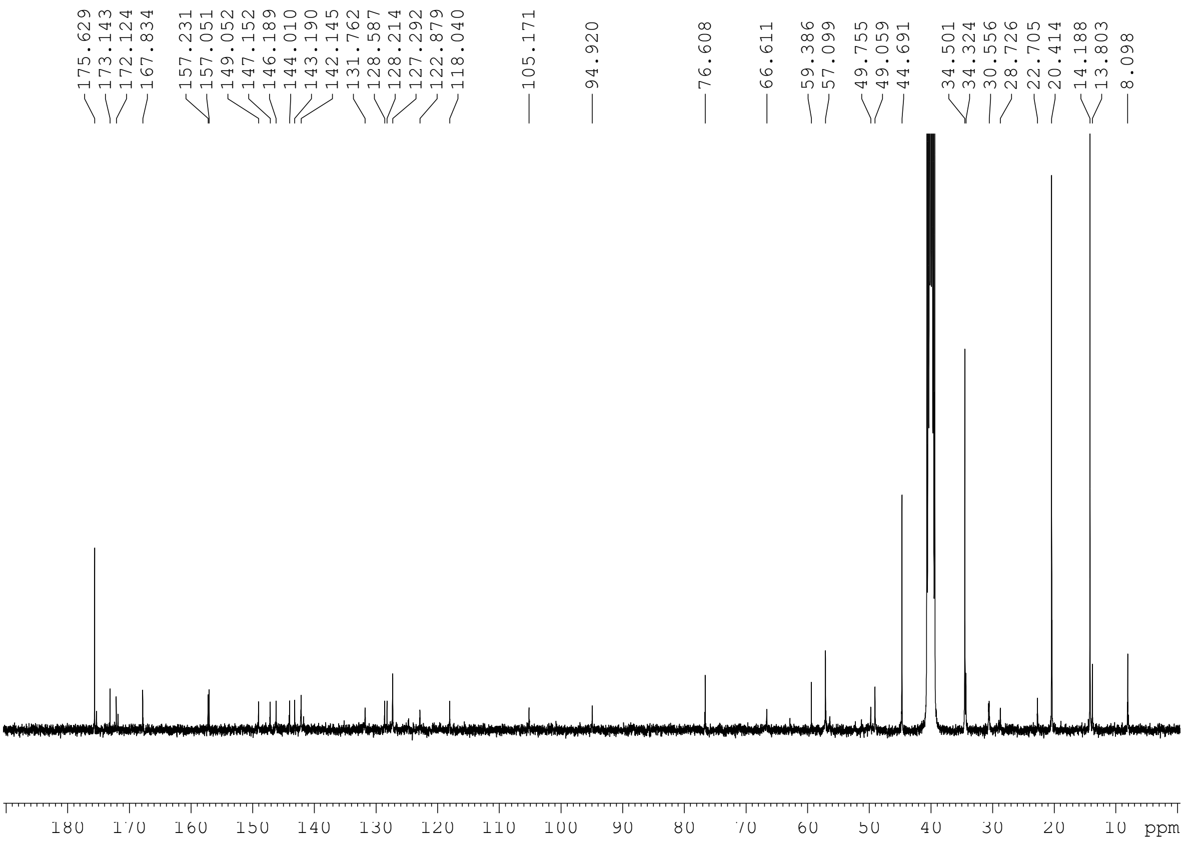

Figure S8. ^{13}C NMR spectrum of compound **8** (101 MHz,DMSO-d_6).

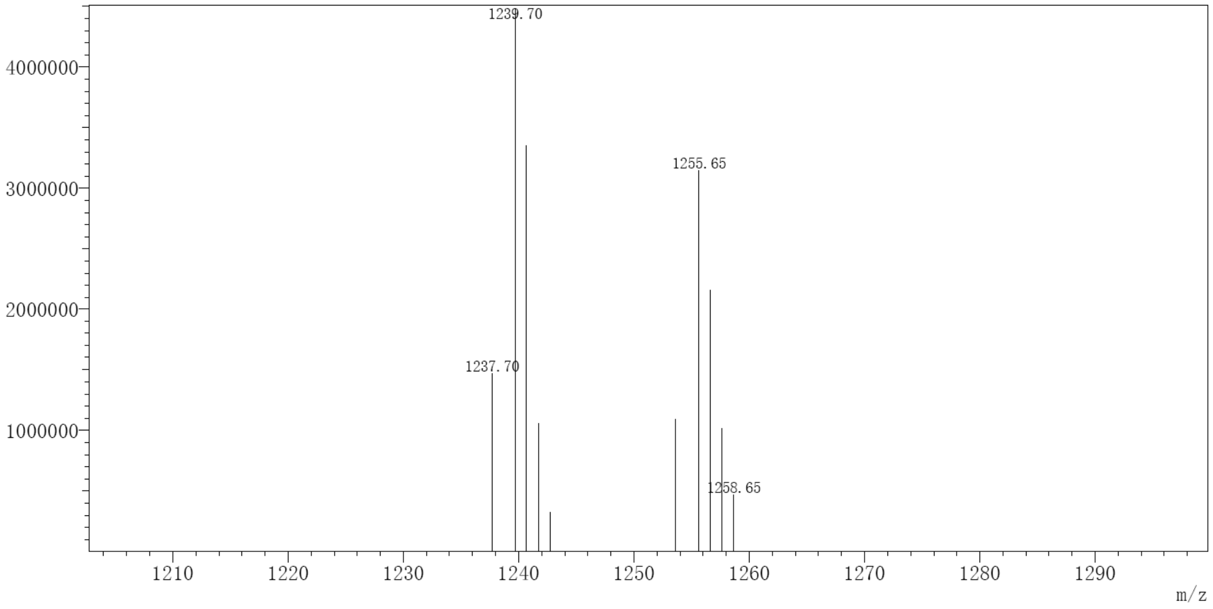

Figure S9. ESI-MS spectrum of compound **8**.

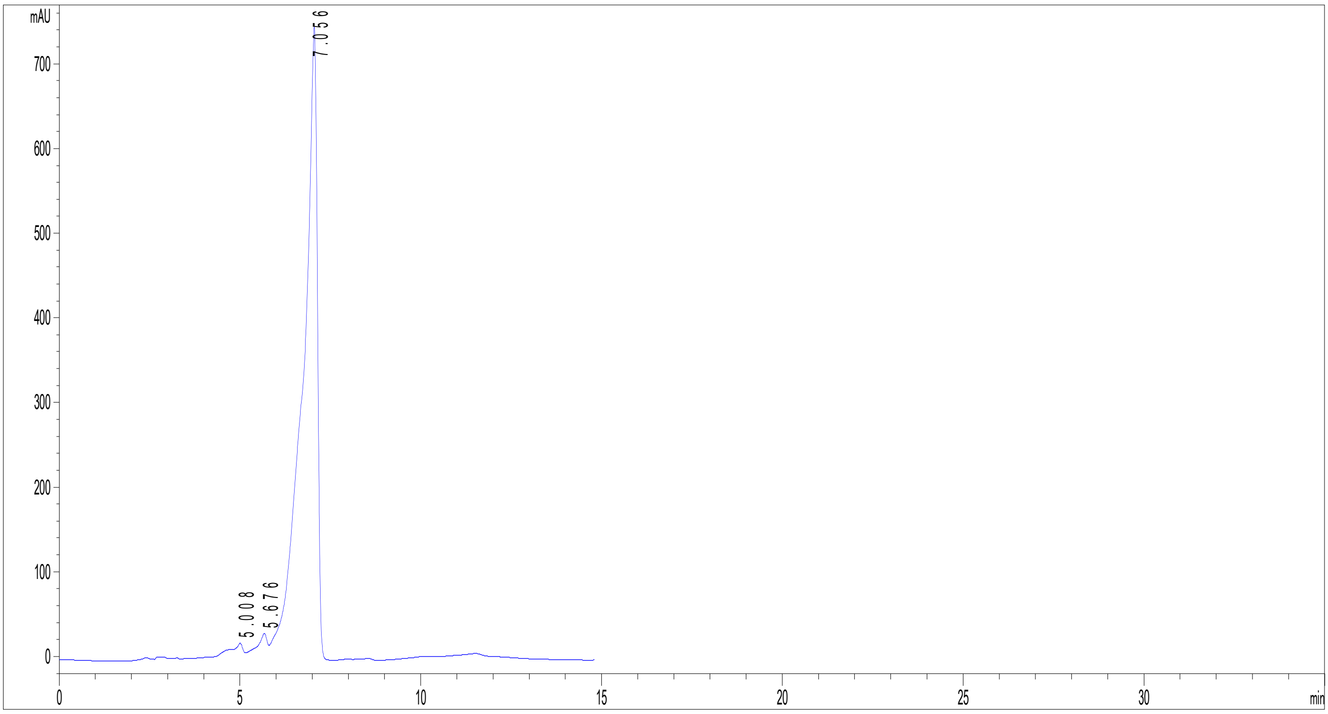

Figure S10. HPLC spectrum of compound **8** (acetonitrile-H_2O=70:30, detection wavelength: 360 nm).

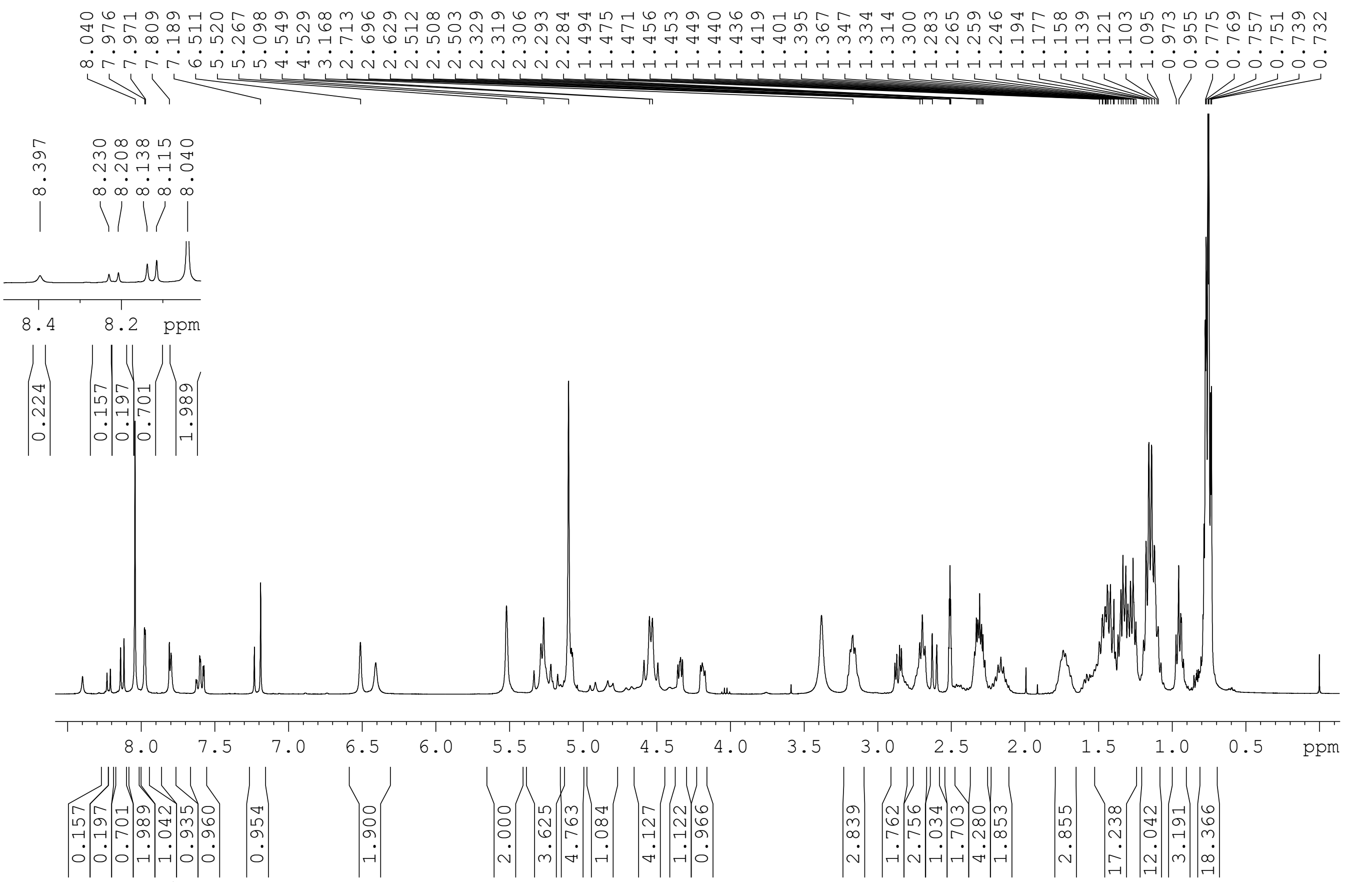

Figure S11. ^{1}H NMR spectrum of compound **9** (400 MHz,DMSO-d_6).

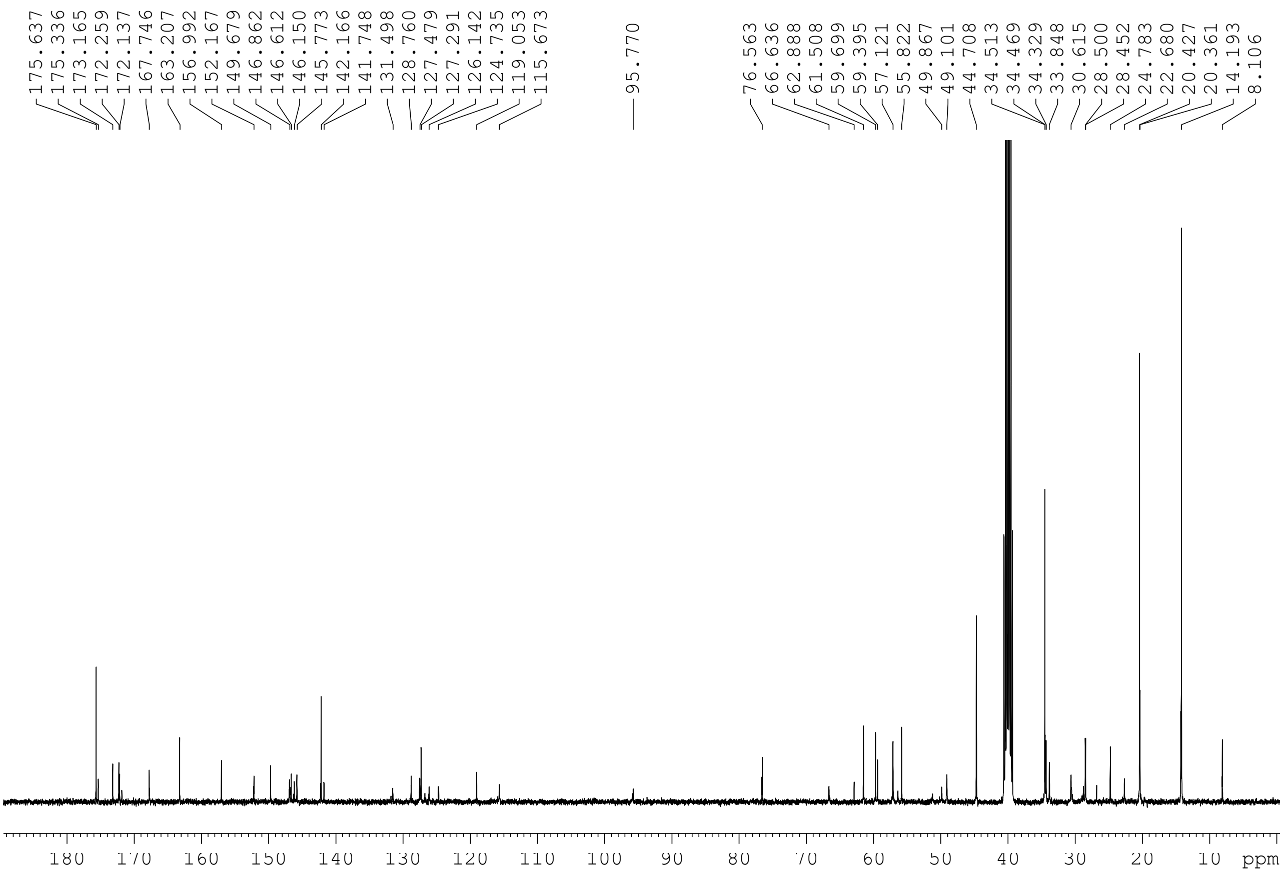

Figure S12. ^{13}C NMR spectrum of compound **9** (101 MHz,DMSO-d_6).

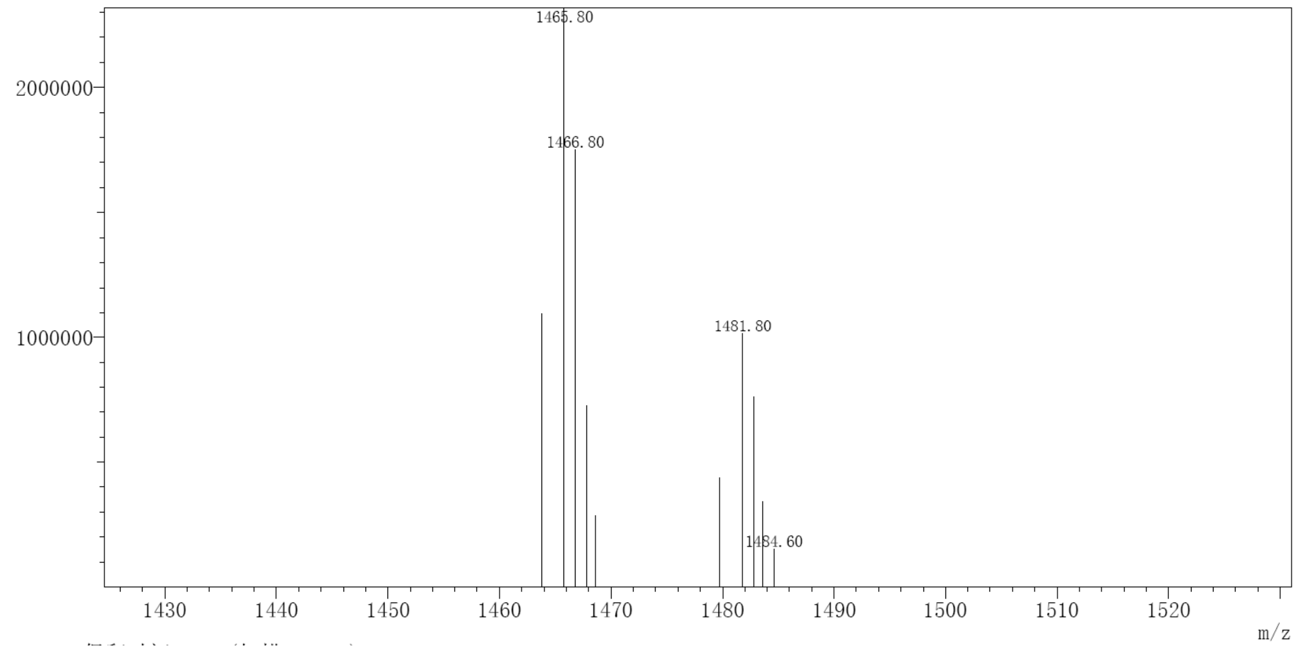

Figure S13. ESI-MS spectrum of compound **9**.

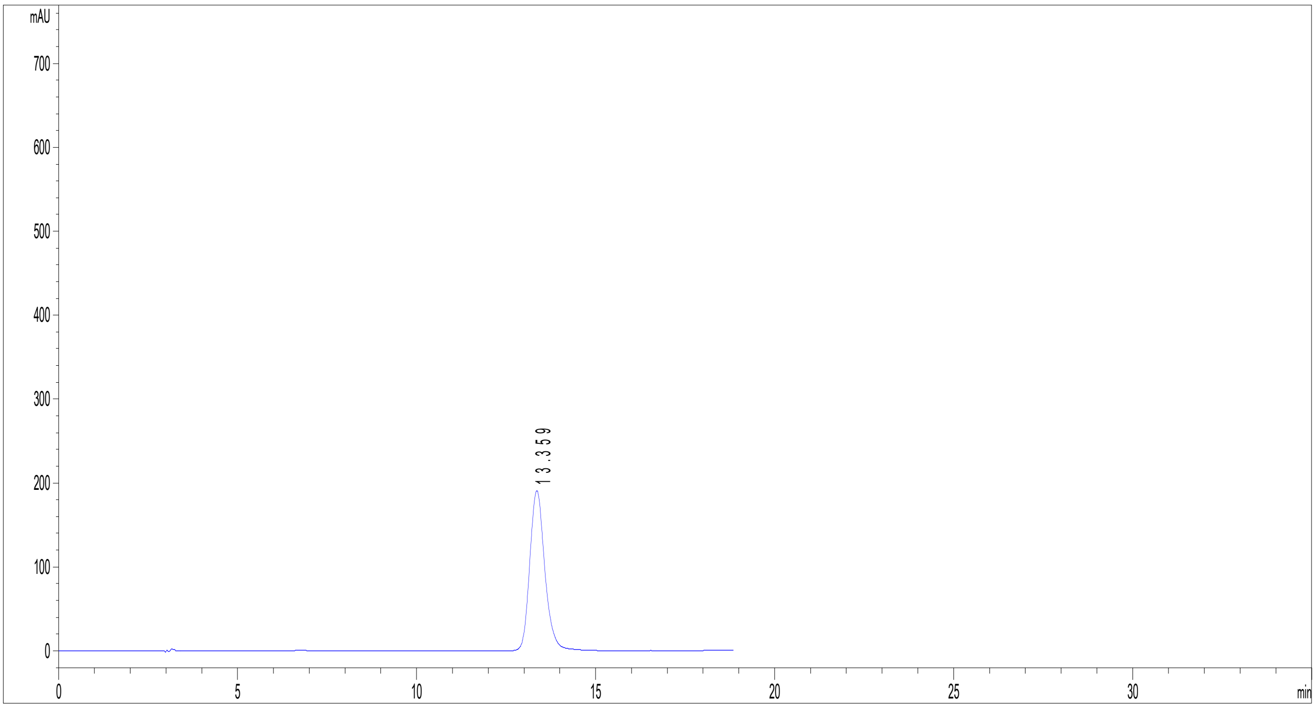

Figure S14. HPLC spectrum of compound **9** (acetonitrile-H_2O=80：20, detection wavelength: 360 nm).

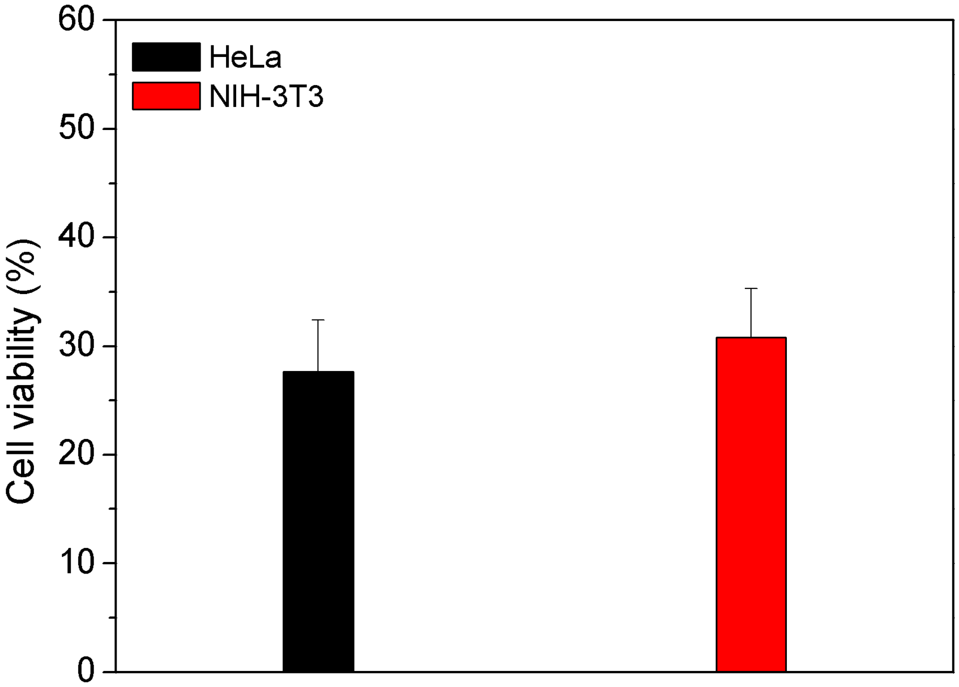

Figure S15. Effects of compound **8** on cell viability of HeLa cells and NIH3T3 cells. Cells were treated with 50 μM of biotin for 48 h and assayed by MTT method.

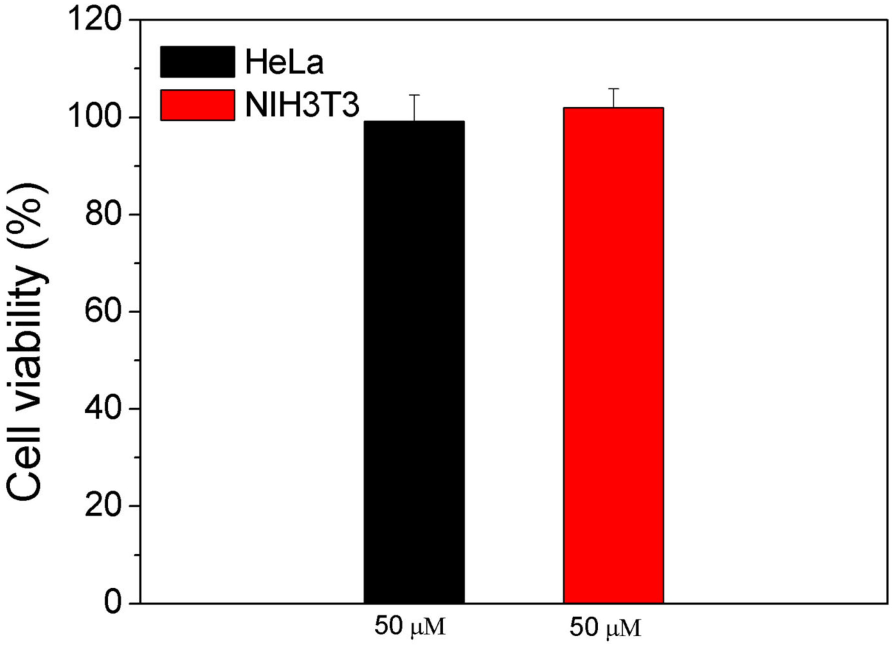

Figure S16. Effects of biotin on cell viability. Cells were treated with 50 μM of biotin for 48 h and assayed by MTT method.

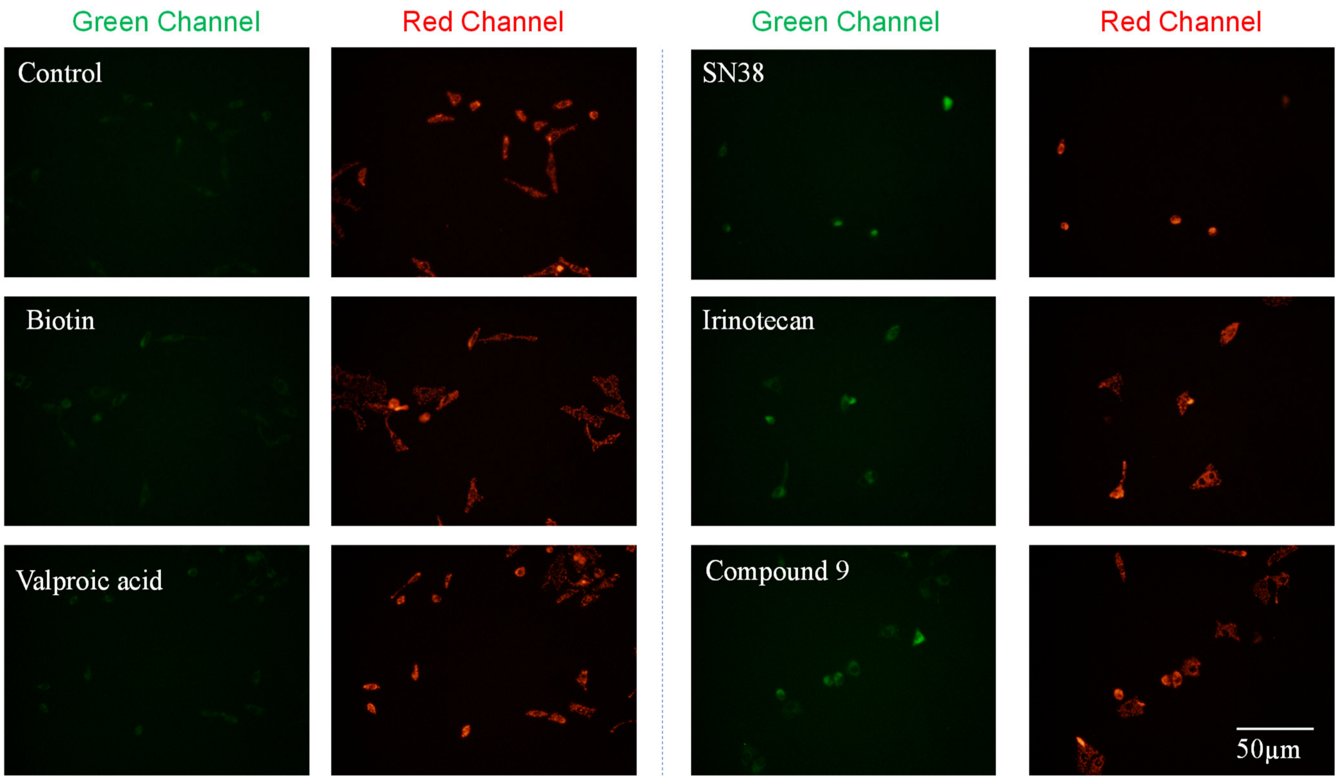

Figure S17. Effects of the tested compounds on mitochondrial membrane potentials. HeLa cells were treated with 2 μM of compounds (or 6 μM of valproic acid) for 24 h and stained with JC-1 probe for determination of mitochondrial membrane potentials using fluorescence microscope.
